# UV Aging Strengthens the Effects of Polyvinyl Chloride Microplastics on Soil Bacterial Community Structure and Predicted Functional Profiles

**DOI:** 10.3390/biology15141181

**Published:** 2026-07-17

**Authors:** Xiaoqing Meng, Yifan Xue, Min Shen, Yu Shen

**Affiliations:** 1 College for Rural Revitalization, Jiangsu Open University, Nanjing 210036, China; shenmin@jsou.edu.cn; 2 College of Ecology and Environment, Nanjing Forestry University, Nanjing 210037, China; xueyifan@163.com (Y.X.); yushen@njfu.edu.cn (Y.S.)

**Keywords:** polyvinyl chloride microplastics, UV aging, soil bacterial community, community structure, predicted functional potential

## Abstract

Soil microplastics undergo aging, but how aging modifies their effects on soil bacterial communities remains unclear. Here, we conducted a 180-day incubation experiment with no PVC (CK), pristine PVC microplastics (IP), and UV-aged PVC microplastics (AP, 0.5%, *w*/*w*). UV aging markedly altered PVC surface properties: roughness increased from approximately 12.9 to 21.8 nm, water contact angle decreased from 91.44° to 82.38°, and the O/C ratio increased from 0.37 to 0.43. Bacterial richness indices were largely unchanged, whereas Shannon diversity decreased under AP, indicating reduced community evenness. Bray–Curtis analysis showed significant community separation among treatments (PERMANOVA: R^2^ = 0.364, *p* = 0.003), with UV aging further altering the trajectory of PVC-induced community reorganization. At the genus level, AP was associated with enrichment of *Methylobacillus* and lower robustness in exploratory co-occurrence network analysis, suggesting a distinct bulk-soil bacterial response compared with IP. Functional prediction further suggested that AP and IP were associated with different predicted pathway profiles, with AP showing higher predicted representation of pathways related to carbon metabolism, respiratory energy metabolism, potential prokaryotic carbon fixation, environmental sensing, cellular maintenance, and antimicrobial-resistance-associated categories, whereas IP was mainly associated with transport- and communication-related predicted functions. These predicted functional patterns require further validation using metagenomic, qPCR, transcriptomic, biochemical, or chemical approaches. Overall, these findings highlight the need to consider the UV aging status of PVC microplastics when evaluating their effects on soil bacterial communities.

## Simple Summary

Microplastics can remain in soil for long periods, during which sunlight and other environmental factors gradually change their surfaces. These aged particles may interact with soil microorganisms differently from newly produced plastics, but this difference is often overlooked when evaluating microplastic effects. In this study, we compared soil without plastic, soil containing pristine polyvinyl chloride microplastics, and soil containing UV-aged polyvinyl chloride microplastics after 180 days of incubation. UV aging made the plastic surface rougher, more oxidized, and less water-repellent. Compared with pristine PVC, UV-aged PVC was associated with a clearer bulk-soil bacterial response, including lower community evenness, higher relative abundance of *Methylobacillus*, and lower robustness in exploratory co-occurrence network analysis. Predicted bacterial functions also differed between pristine and UV-aged PVC, but these results were inferred from 16S rRNA gene-based functional prediction and require further validation. These findings suggest that UV-aged microplastics should not be treated as equivalent to pristine microplastics when evaluating their effects on soil bacterial communities.

## 1. Introduction

Microplastics (MPs), generally defined as plastic particles smaller than 5 mm, have become widespread environmental contaminants across aquatic, atmospheric, and terrestrial systems [1,2]. Soils are now recognized as major reservoirs of MPs, and agricultural soils are particularly vulnerable because plastic mulching, irrigation pipes, compost, sewage sludge, atmospheric deposition, and plastic waste can continuously introduce plastic residues into soil systems [3,4,5]. Because soil microorganisms drive nutrient cycling, organic matter transformation, and plant–soil interactions, MP-induced changes in microbial communities may have important implications for soil ecological functioning [6,7,8].

Beyond their role as inert particles, MPs can act as artificial interfaces in soil. Plastic surfaces provide colonizable microhabitats and can support plastisphere communities that differ from surrounding soil microbial assemblages [9,10]. The formation of these microplastic-associated microbial communities is controlled by polymer type, environmental conditions, and surface properties, including roughness, hydrophobicity, charge, and hardness [10,11,12]. Therefore, microbial responses to MPs should be considered not only as responses to plastic addition, but also as responses to the specific surface interface created by the plastic particles.

In natural environments, MPs are rarely maintained in their pristine state. Weathering and aging processes can change polymer surfaces through photo-oxidation, chain scission, cracking, increased roughness, altered wettability, and formation of oxygen-containing functional groups [13,14]. For PVC specifically, UV irradiation can substantially alter surface morphology and chemistry, including increased carbonyl formation, oxygen-containing functional groups, surface roughening, and leaching behavior [15,16,17]. These UV aging-induced changes may modify bacterial attachment, interfacial selection, and potential particle-associated processes, but most laboratory studies still rely heavily on pristine particles, which may underestimate or misrepresent the ecological effects of environmentally UV-aged MPs [11,18,19].

Microbial functional responses to UV-aged MPs are also insufficiently understood [13,19,20]. MPs can affect soil carbon and nitrogen transformation by changing soil physicochemical and microbial properties, and recent reviews have suggested that MPs may influence soil carbon storage, greenhouse gas emissions, nutrient cycling, and plant-growth-related soil processes [20,21,22]. MPs may also be associated with changes in microbial stress-response and antimicrobial-resistance-related traits, but such effects require direct gene-level validation [23,24]. However, it remains unclear whether UV-aged PVC mainly strengthens the same microbial responses induced by pristine PVC or redirects bacterial community assembly and predicted functional reallocation related to carbon transformation, potential carbon fixation, environmental sensing, cellular maintenance, and antimicrobial-resistance-associated categories.

To address this gap, we conducted a 180-day soil incubation experiment comparing no-plastic control soil, pristine PVC-amended soil, and UV-aged PVC-amended soil. We hypothesized that UV aging would transform PVC into a rougher, more oxidized, and less hydrophobic surface interface, thereby altering bacterial community structure, dominant taxa, co-occurrence network stability, and predicted functional profiles. Specifically, we asked three linked questions: (i) how UV aging changes the surface morphology and chemistry of PVC microplastics; (ii) whether UV-aged PVC induces different bacterial diversity, community composition, dominant genera, and network stability from pristine PVC; and (iii) whether these community changes are accompanied by functional reallocation related to carbon transformation, potential carbon fixation, environmental sensing, cellular maintenance, and antimicrobial-resistance-associated functional potential. Because bacterial communities were analyzed from bulk soil samples, this study evaluates treatment-level soil bacterial responses rather than PVC-attached plastisphere communities. This study provides a more UV aging-relevant basis for evaluating the ecological risks of PVC microplastics in soil environments.

## 2. Materials and Methods

### 2.1. Materials

Poly (vinyl chloride) (PVC) resin was purchased from Sinopec Beijing Yanshan Petrochemical Co., Ltd. (Beijing, China) and supplied as circular disks (3 mm in diameter, 0.5 mm in thickness). To remove surface impurities, the PVC disks were ultrasonically cleaned three times with Milli-Q ultrapure water, dried in a vacuum oven at 45 °C for 48 h, cooled to room temperature, and sealed for storage. Milli-Q water (18.2 MΩ·cm) was produced using a Milli-Q ultrapure water system (Millipore, Bedford, MA, USA).

Topsoil (0–20 cm) was collected from the Baima Experimental Station of Nanjing Agricultural University (119°10′37″ E, 31°37′09″ N), China. The soil was air-dried, manually cleared of plant residues and gravel, gently ground, and passed through a 1 mm sieve. The physicochemical properties of the air-dried soil were as follows: pH (H_2_O) = 6.35, organic carbon = 15.94 g kg^−1^, total nitrogen = 1.15 g kg^−1^, available phosphorus = 105.23 mg kg^−1^, clay = 6.85%, silt = 65.83%, and sand = 27.32%.

### 2.2. Plastic UV Aging Treatment

#### 2.2.1. Estimation of Cumulative UV Dose and Nominal Exposure Duration

Based on data released by the China Meteorological Administration (https://www.cma.gov.cn/), the annual mean cumulative solar radiation in China is approximately 5000 MJ m^−2^, of which ultraviolet (UV) radiation accounts for approximately 7%. Thus, the annual cumulative UV dose was estimated to be approximately 350 MJ m^−2^. A UV lamp (365 nm, 1.0 kW) was used as the light source for the UV aging experiment, and the effective irradiation area of the chamber was 0.3 m^2^. Based on lamp power and irradiation area, the nominal UV dose rate in the chamber was estimated as 12 MJ m^−2^ h^−1^. Accordingly, ~29 h of UV exposure in the chamber was regarded as a nominal one-year natural UV exposure estimate, and ~87 h was regarded as a nominal multi-year natural UV exposure estimate based on cumulative UV dose. This estimate was used only to define an accelerated UV-driven aging scenario and should not be interpreted as an equivalent simulation of complete natural weathering.

#### 2.2.2. UV Aging of Microplastics

To obtain PVC microplastics with different UV aging states, dried PVC disks were evenly spread (without a lid) in glass Petri dishes (10 cm in diameter) at 10 g per dish, with an approximate layer thickness of 1 mm. The dishes were placed in a UV aging chamber and UV-aged under ambient air using a 365 nm UV lamp (1.0 kW), with a fixed and consistent distance between the samples and the light source.

To ensure uniform irradiation, the PVC disks were manually turned once every 4 h. The cumulative UV exposure time was 12 h per day, and the lamp was switched off for the remaining time. The PVC disks were exposed to UV light for 87 h as an accelerated UV-aged treatment [16]. Based on chamber irradiance and annual mean UV radiation estimates, this treatment approximately corresponds to a nominal multi-year natural UV exposure scenario, but it does not fully replicate field weathering conditions. After UV aging, the samples were cooled to room temperature, transferred to amber glass bottles, and stored in a desiccator in the dark for subsequent incubation experiments and physicochemical characterization. Pristine PVC disks were also stored in the dark.

### 2.3. Soil Incubation Experiment

Air-dried soil was thoroughly mixed with PVC microplastics according to the experimental design. Three treatments were established: CK (soil without PVC addition), IP (soil amended with pristine PVC), and AP (soil amended with UV-aged PVC microplastics). Each treatment included three independent biological replicates, with each pot serving as one biological replicate. PVC microplastics were added at 0.5% (*w*/*w*, dry-soil basis). This dose corresponds to 5 g kg^−1^ dry soil and represents a controlled high-exposure PVC-residue scenario rather than a typical average field concentration. The mixture was placed in clean plastic bags, mechanically shaken, and manually stirred for at least 20 min until it appeared homogeneous by visual inspection. Subsequently, 90 g of the treated soil was added to a ceramic pot and gently compacted. Deionized water was added to adjust the soil moisture to 60% of field water-holding capacity (FWHC), and the initial pot weight was recorded. The pots were incubated in a controlled-environment growth chamber under the following conditions: 16 h light at 25 °C and 8 h dark at 20 °C, relative humidity of ~60%, and a light intensity of approximately 250 μmol m^−2^ s^−1^. Pot positions were randomly rearranged every 3 days to minimize positional effects associated with light and air circulation.

During incubation, soil moisture was maintained gravimetrically. Pots were weighed every 2 days, and deionized water was replenished based on weight loss to keep moisture at ~60% FWHC. After 180 days, the incubation was terminated, and soil samples were collected. The soil in each pot was gently removed, thoroughly homogenized, and subsampled randomly. Subsamples were immediately transferred into sterile centrifuge tubes, flash-frozen in liquid nitrogen, and stored at −80 °C for subsequent DNA extraction and microbial community analysis. All nine soil samples were successfully subjected to 16S rRNA gene amplicon sequencing and included in the downstream analyses.

### 2.4. Microbial Diversity Analysis

Total soil DNA was extracted using the E.Z.N.A.^®^ Soil DNA Kit (Omega Bio-tek, Norcross, GA, USA) following the manufacturer’s instructions. DNA integrity was assessed by 1% agarose gel electrophoresis, and DNA concentration and purity were measured using a NanoDrop 2000 spectrophotometer (Thermo Fisher Scientific, Waltham, MA, USA).

Bacterial community composition was assessed by PCR amplification of the V4–V5 region of the 16S rRNA gene using primers 515F (5′-GTGCCAGCMGCCGCGG-3′) and 907R (5′-CCGTCAATTCMTTTRAGTTT-3′) [25]. The PCR program was as follows: initial denaturation at 94 °C for 4 min; 25 cycles of 94 °C for 30 s, 55 °C for 30 s, and 72 °C for 60 s; and a final extension at 72 °C for 10 min, followed by holding at 4 °C. Three technical PCR replicates were performed for each soil sample, and PCR products from the same sample were pooled in equal volumes.

Pooled amplicons were separated on a 2% agarose gel, and target bands were excised and purified using the AxyPrep DNA Gel Extraction Kit (Axygen Biosciences, Union City, CA, USA). Purified DNA was quantified using a Qubit^®^ 3.0 fluorometer (Life Technologies/Invitrogen, Calsbad, CA, USA). Sequencing libraries were constructed using the NEXTflex™ Rapid DNA-Seq Kit (Bioo Scientific, Austin, TX, USA), including adapter ligation, magnetic-bead removal of adapter dimers, PCR enrichment, and bead-based purification. After passing quality control, paired-end sequencing (PE300) was conducted on an Illumina MiSeq platform (Illumina, San Diego, CA, USA) [26]. Library preparation and sequencing were performed by Shanghai LingEn Biotechnology Co., Ltd. (Shanghai, China). Raw reads were quality-filtered prior to downstream bioinformatics analyses.

### 2.5. Microplastic Characterization

Surface morphological changes in PVC microplastics before and after UV aging were examined by scanning electron microscopy (SEM; Regulus 8100, Hitachi, Tokyo, Japan). Three-dimensional surface topography was characterized using atomic force microscopy (AFM; MultiMode 8, Bruker, Billerica, MA, USA), and surface roughness parameters were calculated accordingly. The hydrophilic/hydrophobic properties of PVC microplastics were evaluated using a contact angle meter (OCA20, Dataphysics, Filderstadt, Germany). Functional group changes in PVC microplastics before and after UV aging in different treatments were identified by attenuated total reflectance Fourier transform infrared spectroscopy (ATR-FTIR; Nicolet iS50, Thermo Fisher Scientific, Waltham, MA, USA). The surface chemical structure and elemental composition of PVC microplastics before and after UV aging were further analyzed by X-ray photoelectron spectroscopy (XPS; ESCALAB 250Xi, Thermo Fisher Scientific, Waltham, MA, USA).

### 2.6. Data Processing and Statistical Analysis

Raw paired-end reads were processed using the Biozeron pipeline (v10). Briefly, reads were demultiplexed according to barcodes and primers, quality-filtered, merged, and checked for chimeras. Detailed filtering parameters are provided in Supplementary Methods S1. OTUs were clustered at 97% sequence similarity using UPARSE, and taxonomic assignment of representative OTU sequences was performed using the RDP classifier against the SILVA database with a confidence threshold of 0.7. A total of 11,454 raw OTUs were obtained, and 7400 OTUs were retained after filtering.

The OTU table was rarefied to the minimum sequencing depth across all samples before alpha-diversity analysis. Alpha-diversity indices, including Chao1, ACE, Shannon, and Observed species, were calculated using the vegan package in R. Differences among treatments were tested by one-way ANOVA followed by Tukey’s HSD test. Different lowercase letters in the figures indicate significant differences among treatments at *p* < 0.05. Detailed statistical outputs for the main treatment comparisons, including test statistics, degrees of freedom, effect sizes where applicable, letter groupings, and Tukey-adjusted *p* values, are provided in Supplementary Table S1A.

Beta diversity was evaluated based on Bray–Curtis distances. PCoA was performed to visualize differences in bacterial community composition, and PERMANOVA was conducted using the adonis function in the vegan package with 999 permutations [27]. Within-group dispersion was evaluated based on within-treatment Bray–Curtis group distances, and treatment differences were tested by one-way ANOVA followed by Tukey’s HSD test. The ellipses in the PCoA plots represent 95% confidence ellipses.

Co-occurrence networks were constructed at the OTU level using the filtered OTU abundance table. Pairwise associations among OTUs were calculated using Spearman correlation analysis, and edges were retained when Spearman’s |ρ| > 0.6 and *p* < 0.05. The number of nodes and edges retained in each treatment network is provided in Supplementary Table S1B. Network-related indices, including cohesion [28], vulnerability, and robustness, were calculated to compare treatment-dependent changes in bacterial co-occurrence patterns. Given the limited number of biological replicates within each treatment, the network results were interpreted as exploratory indicators of co-occurrence patterns rather than direct evidence of microbial interactions.

Predicted functional profiles were inferred from 16S rRNA gene data using Tax4Fun2 [29]. Predicted KEGG pathways were summarized at Level 2 and Level 3. For heatmap visualization, pathway relative abundance values were Z-score transformed by row. These results were interpreted as predicted functional tendencies rather than direct measurements of functional genes, transcripts, proteins, or metabolic activities.

## 3. Results and Discussion

### 3.1. Material Characterization

**Figure 1:**
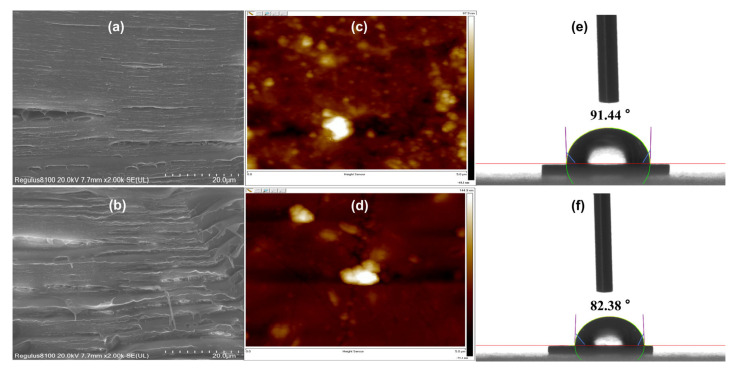
Surface morphology, roughness, and wettability of pristine and UV-aged PVC microplastics. (**a**) SEM image of pristine PVC microplastics. (**b**) SEM image of PVC microplastics after 87 h UV aging. (**c**) AFM height map of pristine PVC microplastics (5 μm × 5 μm). (**d**) AFM height map of PVC microplastics after 87 h UV aging (5 μm × 5 μm). (**e**) Water contact angle image of pristine PVC microplastics. (**f**) Water contact angle image of PVC microplastics after 87 h UV aging.

SEM, AFM, and water contact angle analyses showed that UV aging substantially altered the surface morphology, roughness, and wettability of PVC microplastics (Figure 1). Pristine PVC showed a relatively intact and continuous surface with oriented banded/lamellar features and only shallow grooves (Figure 1a). After UV aging, the surface became visibly damaged, with disrupted lamellae, interlayer cracks, step-like delamination, through-going fractures, and localized pores (Figure 1b). Similar surface roughening, crack formation, and flake-like deterioration have been reported for UV-aged PVC microplastics under UV irradiation, indicating that these changes are characteristic features of PVC UV aging [15,16,23]. More generally, solar UV-driven photo-oxidation can increase the susceptibility of plastic debris to subsequent fragmentation, which is consistent with the brittle surface deterioration observed here [30,31].

AFM further confirmed the UV aging-induced increase in nanoscale surface heterogeneity. Compared with pristine PVC, which showed limited height variation, UV-aged PVC displayed more pronounced ridge-valley structures and larger particulate protrusions (Figure 1c,d). The quantified roughness also increased after UV aging, with Rq rising from approximately 12.9 nm in pristine PVC to approximately 21.8 nm in UV-aged PVC. Meanwhile, the water contact angle decreased from 91.44° to 82.38° after UV aging (Figure 1e,f), indicating that the PVC surface became less hydrophobic. Together, these results show that UV aging converted PVC microplastics from a relatively smooth and hydrophobic particle surface into a rougher and more wettable interface.

**Figure 2:**
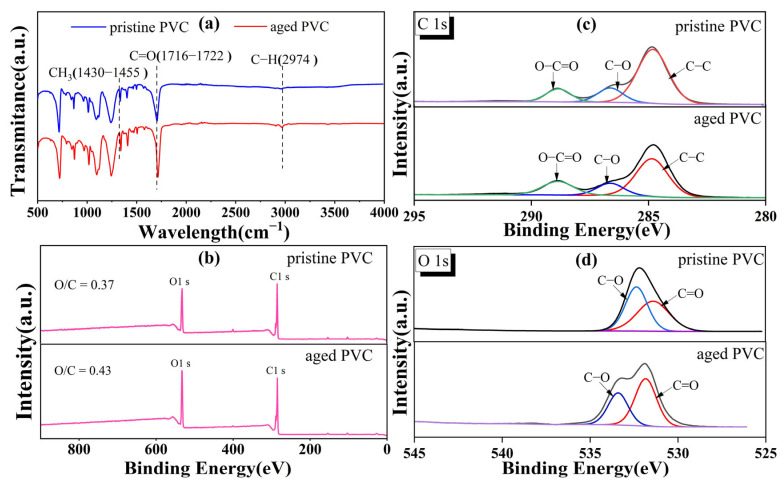
Chemical characterization of pristine and UV-aged PVC microplastics. (**a**) ATR-FTIR spectra. (**b**) XPS survey spectra showing changes in the O/C ratio. (**c**) High-resolution C 1s spectra. (**d**) High-resolution O 1s spectra.

ATR-FTIR and XPS analyses further demonstrated that these physical changes were accompanied by surface chemical oxidation (Figure 2). In the ATR-FTIR spectra, the band at 1716–1722 cm^−1^, assigned to C=O stretching, became more evident after UV aging, indicating the formation of oxygen-containing functional groups during UV exposure (Figure 2a). Previous work on PVC UV aging also reported increased oxygen-containing functional groups and a higher carbonyl index with increasing UV exposure, supporting the interpretation that UV irradiation promotes oxidative modification of PVC surfaces [15,32,33,34,35]. XPS results were consistent with the FTIR evidence: the O/C ratio increased from 0.37 in pristine PVC to 0.43 in UV-aged PVC (Figure 2b), and high-resolution C 1s spectra showed increased contributions of oxygenated carbon species, including C-O and O-C=O, after UV aging (Figure 2c). The corresponding O 1s spectra also indicated changes in C-O and C=O components (Figure 2d). These results confirm that UV aging enhanced the surface oxidation of PVC microplastics.

Overall, UV aging transformed PVC microplastics into a rougher, more oxidized, and less hydrophobic material surface, as indicated by surface cracking, delamination, increased roughness, reduced water contact angle, and enhanced oxygen-containing functional groups. These UV aging-induced changes provide the material basis for interpreting the subsequent bulk-soil bacterial responses, although they do not directly demonstrate PVC-surface colonization or biofilm formation.

### 3.2. Alpha Diversity

**Figure 3:**
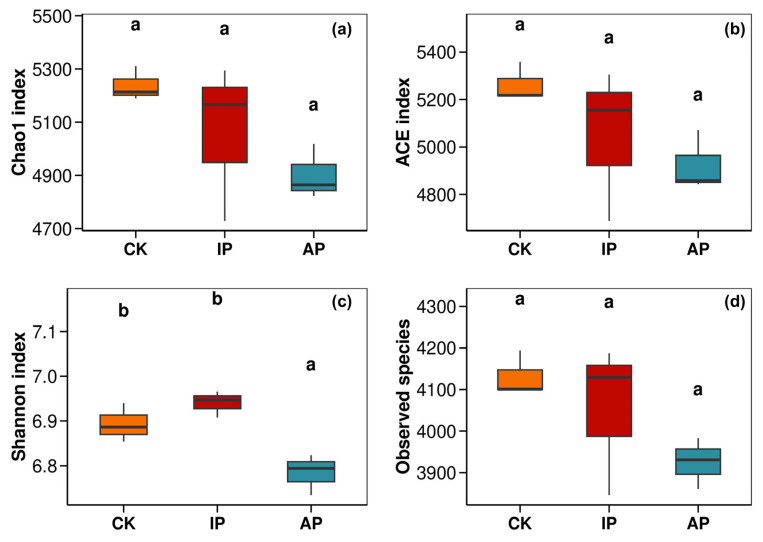
Alpha diversity of soil bacterial communities under different treatments. (**a**) Chao1 index. (**b**) ACE index. (**c**) Shannon index. (**d**) Observed species. Different lowercase letters above the bars indicate significant differences among treatments at *p* < 0.05 based on one-way ANOVA followed by Tukey’s HSD test; treatments sharing the same letter are not significantly different. IP, soil amended with pristine PVC microplastics; AP, soil amended with UV-aged PVC microplastics.

Observed species, Chao1, and ACE primarily reflect richness-related information within bacterial communities, whereas the Shannon index incorporates both richness and evenness through taxon abundance distribution [36,37,38]. As shown in Figure 3, CK, IP, and AP were comparable in Chao1, ACE, and Observed species, suggesting that PVC addition did not markedly change bacterial richness under the present incubation conditions (Figure 3a,b,d). In contrast, the Shannon index was lower in AP than in CK and IP, whereas CK and IP were similar (Figure 3c). This result indicates that UV-aged PVC mainly changed the evenness and relative abundance distribution of the bacterial community, rather than causing a broad loss of bacterial taxa.

The reduced Shannon index under AP, together with the largely unchanged richness indices, indicates a shift in the relative abundance distribution rather than broad taxon loss. Given that plastic surface properties and environmental conditions can influence bacterial adhesion and microplastic-associated processes [8,39,40,41,42], UV aging-induced changes in PVC surface properties and possible soluble degradation products may have contributed to this bulk-soil community response. However, because PVC-attached communities were not separately analyzed, this result should not be taken as direct evidence of PVC-surface colonization or biofilm assembly.

### 3.3. Beta Diversity

**Figure 4:**
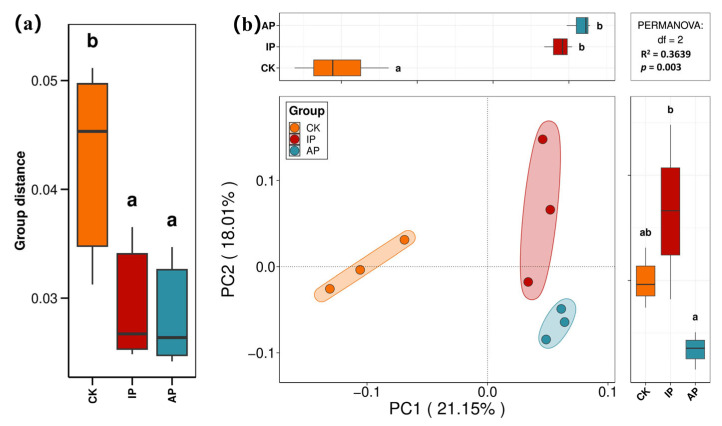
Beta diversity of soil bacterial communities under different treatments based on Bray–Curtis distance. (**a**) Group distance based on within-treatment Bray–Curtis distances. (**b**) Principal coordinates analysis (PCoA). Different lowercase letters indicate significant differences among treatments for the corresponding boxplots at *p* < 0.05 based on Tukey’s HSD test. Ellipses indicate 95% confidence intervals. CK, control soil; IP, soil amended with pristine PVC microplastics; AP, soil amended with UV-aged PVC microplastics.

The within-group distance analysis showed that both PVC-amended treatments had significantly lower group distances than CK, whereas IP and AP did not differ significantly from each other (Figure 4a). This result indicates that PVC addition, regardless of UV aging status, reduced within-treatment community dispersion and produced a more consistent bacterial community configuration across replicates. Because distance-based tests of multivariate dispersion can be used to evaluate differences in within-group variability, dispersion results should be interpreted together with ordination and PERMANOVA results [43,44].

The PCoA based on Bray–Curtis distance showed separation among CK, IP, and AP, with PC1 and PC2 explaining 21.15% and 18.01% of the total variation, respectively (Figure 4b). PERMANOVA confirmed a significant treatment effect on bacterial community composition (df = 2, R^2^ = 0.3639, *p* = 0.003), indicating that treatments explained approximately 36% of the variation in the Bray–Curtis distance matrix. CK was separated from the two PVC-amended treatments mainly along PC1, whereas IP and AP were separated mainly along PC2, suggesting that PVC addition drove the main community shift and UV aging further altered the direction of this response. Together with the lower within-group distances in PVC-amended soils, these results indicate a two-level response: PVC addition produced a more consistent bacterial community configuration relative to CK, and UV aging further differentiated the AP response from IP. Because bacterial adhesion and microplastic-associated processes can be influenced by plastic surface properties, polymer type, and environmental conditions [9,10,11,12,36,37], the UV aging-induced changes described in Section 3.1 may have modified local soil microenvironments around PVC particles. Thus, UV aging contributed to a distinct bulk-soil bacterial community response rather than simply amplifying the effect of pristine PVC.

### 3.4. Microbial Community Analysis

**Figure 5:**
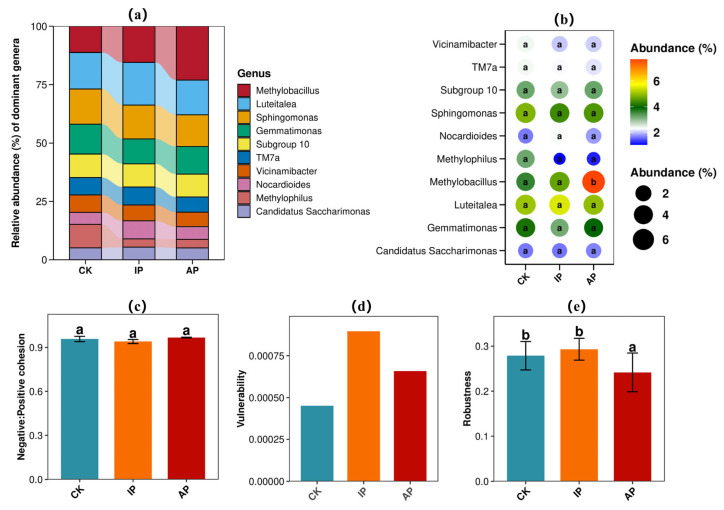
Dominant bacterial genera and co-occurrence network stability under different treatments. (**a**) Stacked bar plot showing the relative abundance of the ten most abundant genera. (**b**) Bubble plot showing the relative abundance of each dominant genus across treatments. (**c**) Negative–positive cohesion. (**d**) Vulnerability. (**e**) Robustness. In panels (**b**,**c**,**e**), different lowercase letters indicate significant differences among treatments at *p* < 0.05 based on one-way ANOVA followed by Tukey’s HSD test; treatments sharing the same letter are not significantly different. CK, control soil; IP, soil amended with pristine PVC; AP, soil amended with UV-aged PVC microplastics.

The relative abundances of the top 10 genera are shown in Figure 5a,b. Across CK, IP, and AP, the dominant genera mainly included *Methylobacillus*, *Luteitalea*, *Sphingomonas*, *Gemmatimonas*, Subgroup 10, TM7a, *Vicinamibacter*, *Nocardioides*, *Methylophilus*, and *Candidatus Saccharimonas*. Their overall profiles were broadly similar among treatments, indicating that PVC addition did not broadly restructure the dominant bacterial genera after 180 d of incubation. However, *Methylobacillus* was significantly more abundant in AP than in CK and IP, making it the clearest genus-level response to UV-aged PVC (Figure 5b). Together with the decrease in Shannon diversity under AP, this result indicates a targeted genus-level shift rather than broad restructuring of the dominant bacterial genera.

The enrichment of *Methylobacillus* may be associated with the altered properties of UV-aged PVC and the methylotrophic potential of this genus, but this explanation remains hypothetical because PVC-attached communities and C1-containing degradation products were not directly analyzed. *Methylobacillus* includes methylotrophic taxa, and *Methylobacillus flagellatus* KT is an obligate methanol and methylamine utilizer; meanwhile, sunlight-driven plastic photodegradation can generate dissolved organic carbon [45,46,47]. Thus, the higher relative abundance of *Methylobacillus* under AP may reflect a combined but unverified effect of altered local particle-associated microhabitats and potential C1-containing compounds generated during PVC UV aging.

Network stability metrics further showed that UV-aged PVC affected bacterial co-occurrence stability (Figure 5c–e). Negative–positive cohesion did not differ significantly among treatments, and vulnerability was numerically highest in IP and intermediate in AP. In contrast, robustness was significantly lower in AP than in CK and IP, whereas CK and IP did not differ significantly. Community cohesion quantifies microbial community connectivity, and network robustness reflects the persistence of microbial networks after simulated node removal [28,48,49]. Therefore, the reduced robustness under AP should be interpreted as an exploratory signal of altered co-occurrence patterns rather than direct evidence of changed microbial interactions. Overall, UV-aged PVC was associated with a clearer genus-level response and lower exploratory network robustness than pristine PVC.

### 3.5. Tax4Fun2 Functional Prediction

**Figure 6:**
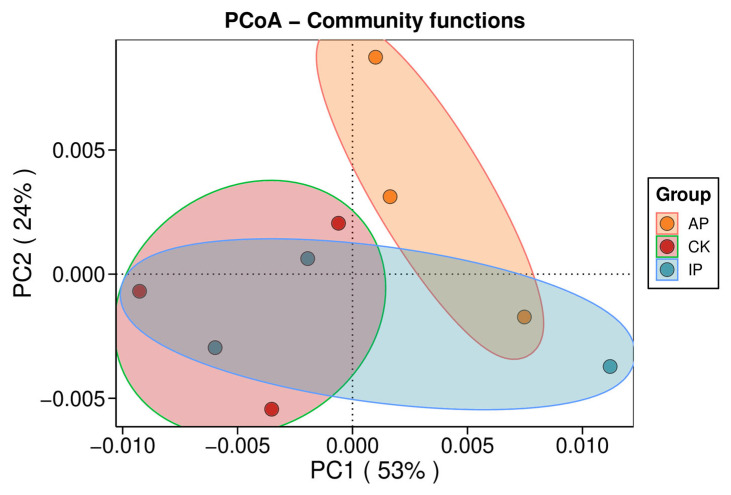
Principal coordinates analysis (PCoA) of Tax4Fun2-predicted KEGG functional profiles of soil bacterial communities under different treatments. CK, control soil; IP, soil amended with pristine PVC microplastics; AP, soil amended with UV-aged PVC microplastics.

The PCoA based on Tax4Fun2-predicted KEGG profiles showed that PC1 and PC2 explained 53% and 24% of the total variation, respectively, accounting for 77% cumulatively (Figure 6). CK was mainly distributed on the negative side of PC1, whereas AP shifted toward the positive direction of PC2. IP showed a broader distribution and partly overlapped with both CK and AP. This pattern suggests that UV-aged PVC was associated with a clearer shift in predicted functional structure than pristine PVC, which is consistent with the beta-diversity results showing that UV aging changed the trajectory of PVC-induced bacterial community reorganization.

**Figure 7:**
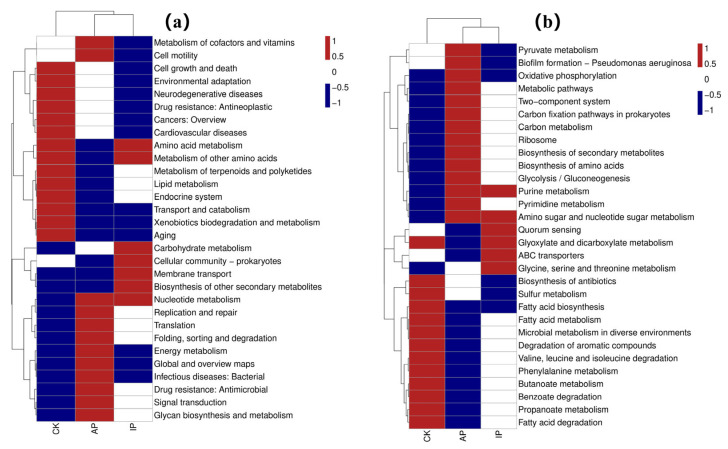
Tax4Fun2-predicted KEGG functional composition of soil bacterial communities under different treatments. (**a**) Heatmap of KEGG Level 2 pathways. (**b**) Heatmap of KEGG Level 3 pathways. CK, control soil; IP, soil amended with pristine PVC microplastics; AP, soil amended with UV-aged PVC microplastics.

At KEGG Level 2, AP showed higher predicted representation of energy metabolism, signal transduction, replication and repair, translation, folding, sorting and degradation, nucleotide metabolism, glycan biosynthesis and metabolism, cell motility, and metabolism of cofactors and vitamins (Figure 7a). In contrast, IP showed relatively higher predicted representation of carbohydrate metabolism, membrane transport, cellular community—prokaryotes, and biosynthesis of other secondary metabolites, whereas CK showed higher predicted representation of transport and catabolism, xenobiotics biodegradation and metabolism, lipid metabolism, and metabolism of terpenoids and polyketides. These results indicate that pristine and UV-aged PVC were associated with different predicted functional profiles: pristine PVC was more associated with predicted transport-, exchange-, and communication-related pathways, whereas UV-aged PVC was more associated with predicted pathways related to energy turnover, signal regulation, genetic information processing, and cellular maintenance.

At KEGG Level 3, AP showed higher predicted representation of carbon metabolism, pyruvate metabolism, glycolysis/gluconeogenesis, oxidative phosphorylation, carbon fixation pathways in prokaryotes, two-component system, ribosome, biosynthesis of amino acids, purine metabolism, pyrimidine metabolism, amino sugar and nucleotide sugar metabolism, and biofilm-related pathway categories (Figure 7b). These predicted pathways suggest that UV-aged PVC may be associated with possible shifts in central carbon transformation, respiratory energy metabolism, environmental sensing, and cellular biosynthesis. However, these patterns were inferred from 16S rRNA gene-based functional prediction and should not be interpreted as direct evidence of actual metabolic activity, carbon fixation, or biofilm formation.

These predicted functional patterns may be related to the UV aging-induced surface changes described in Section 3.1. The rougher, more oxidized, and less hydrophobic AP surface may have altered local particle-associated microhabitats and bulk-soil bacterial community structure. Soil microorganisms are key drivers of soil carbon and nitrogen transformation, and microplastics can influence soil carbon and nitrogen cycling by altering soil physicochemical and microbial properties [6,20,50]. Therefore, the higher predicted representation of carbon- and energy-metabolism-related pathways under AP may reflect changes in bacterial community composition and inferred functional potential, rather than directly measured changes in soil carbon transformation or energy metabolism.

The AP-associated increases in predicted carbon metabolism, pyruvate metabolism, glycolysis/gluconeogenesis, and oxidative phosphorylation were consistent with the selective enrichment of *Methylobacillus* observed in Figure 5. However, the observed result was the higher relative abundance of *Methylobacillus* in AP, whereas the following explanation remains hypothetical. *Methylobacillus* includes methylotrophic taxa, and *Methylobacillus flagellatus* KT can use methanol and methylamine as carbon and energy sources [45]. Sunlight-driven plastic photodegradation can generate dissolved organic carbon, and PVC degradation can produce low-molecular-weight acidic compounds such as formic acid and acetic acid [46,47,51]. Thus, *Methylobacillus* enrichment in AP may be associated with altered local particle-associated microhabitats or potential C1-containing compounds generated during PVC UV aging, but dissolved organic carbon, methanol, organic acids, and PVC-attached communities were not directly measured. In addition, AP showed higher predicted representation of carbon fixation pathways in prokaryotes, glycan biosynthesis and metabolism, and amino sugar and nucleotide sugar metabolism. These pathways may be related to inferred carbon fixation potential and microbial surface-associated carbohydrate processes [52,53,54,55], but they do not provide direct evidence of actual carbon fixation, EPS production, soil aggregation, nutrient retention, or biofilm matrix formation. Therefore, these predicted functional tendencies require further validation.

Predicted antimicrobial-resistance-associated categories also differed among treatments. At KEGG Level 2, AP showed a higher predicted representation of Drug resistance: Antimicrobial than CK and IP. At KEGG Level 3, AP also showed higher predicted representation of two-component system and biofilm-related pathway categories, suggesting possible changes in stress-response-associated predicted functional profiles. However, these results should not be interpreted as evidence of ARG enrichment, ARG transfer, or enhanced antimicrobial resistance activity, because ARGs were not directly quantified and no metagenomic or qPCR validation was performed. The specific factors triggering these predicted categories cannot be identified from the present data and may involve changes in community composition, local microenvironmental conditions, or other unmeasured processes.

Overall, pristine and UV-aged PVC induced different endpoint responses in the bulk-soil bacterial community. Pristine PVC was mainly associated with predicted transport- and communication-related functions, whereas UV-aged PVC was associated with lower Shannon diversity, higher relative abundance of *Methylobacillus*, lower exploratory network robustness, and shifts in predicted pathways related to carbon and energy metabolism, environmental sensing, cellular maintenance, and antimicrobial-resistance-associated categories. However, these functional patterns were inferred from 16S rRNA gene-based prediction and should not be interpreted as direct evidence of functional activity, EPS formation, ARG enrichment, or biofilm development.

It should be noted that bacterial community analysis was performed using DNA extracted from bulk soil samples containing both soil particles and PVC microplastics; therefore, the results represent treatment-level bulk-soil bacterial responses and do not distinguish PVC-attached communities from non-attached soil communities. Because the incubated PVC particles were not retained, endpoint SEM, AFM, or biofilm-specific analyses could not be performed, and direct evidence for PVC-surface colonization or biofilm assembly was not obtained. Similarly, antimicrobial-resistance-associated categories were inferred only from predicted functional profiles, and ARGs were not directly quantified. Thus, this study cannot determine whether PVC microplastics triggered ARG enrichment or identify the specific drivers of these predicted resistance-associated categories. In addition, the UV aging treatment represents an accelerated UV-driven scenario rather than a full simulation of natural weathering, and the single endpoint sampling after 180 days cannot resolve when the observed bacterial shifts emerged or whether they were transient or stable. Endpoint soil physicochemical properties and PVC leachates were not chemically characterized; therefore, indirect effects mediated by soil chemistry or soluble degradation products cannot be separated from responses associated with PVC aging-induced surface changes. Future studies should combine time-series sampling, separated PVC-attached and surrounding-soil microbial analyses, endpoint surface imaging, biofilm-specific staining, metagenomic or qPCR validation, and leachate or soil-chemistry measurements.

## 4. Conclusions

UV aging transformed PVC microplastics into a rougher, more oxidized, and less hydrophobic material surface and was associated with bulk-soil bacterial responses distinct from those induced by pristine PVC. Although bacterial richness remained largely unchanged, UV-aged PVC reduced community evenness, altered community composition, enriched *Methylobacillus*, and decreased robustness in the exploratory co-occurrence network analysis. Functional prediction suggested that pristine and UV-aged PVC were associated with different predicted pathway profiles, but these results require further validation using metagenomic, qPCR, transcriptomic, biochemical, enzyme-activity, or chemical approaches. Because bacterial communities were analyzed from bulk soil rather than separated PVC-attached fractions, the observed microbial changes should be interpreted as bulk-soil community responses rather than direct evidence of plastisphere or biofilm development. In addition, because endpoint soil physicochemical properties, PVC leachates, and soluble degradation products were not characterized, the relative contributions of soil-chemistry-mediated effects, PVC surface changes, and soluble compounds could not be distinguished. Overall, these findings indicate that the UV aging status of PVC microplastics should be considered when evaluating their effects on soil bacterial communities.

## Data Availability

The raw 16S rRNA gene sequencing data generated in this study have been deposited in the Genome Sequence Archive in National Genomics Data Center, China National Center for Bioinformation, under accession number CRA044434. Other data supporting the findings of this study are available within the article.

## Supplementary Materials

The following supporting information can be downloaded at: https://www.mdpi.com/article/10.3390/biology15141181/s1. Supplementary Methods S1. Detailed 16S rRNA gene amplicon data processing parameters. Table S1A. Statistical details for the main treatment comparisons shown in Figure 3, Figure 4 and Figure 5. Table S1B. Co-occurrence network construction parameters.
