# Supplementary material for: UV Aging Strengthens the Effects of Polyvinyl Chloride Microplastics on Soil Bacterial Community Structure and Predicted Functional Profiles
Source: Biology (Basel). 2026 Jul 17;15(14):1181. doi: 10.3390/biology15141181 (PMC13403993; doi:10.3390/biology15141181)
Supplement: Supplementary file 1 [file biology-15-01181-s001.zip › biology-4402113-supplementary.pdf]

Supplementary Methods S1. Detailed 16S rRNA gene amplicon data processing parameters

Raw paired-end reads were demultiplexed according to barcodes and primers, and sequence orientation was corrected according to barcode and primer directions. Low-quality bases at the 3′ end were trimmed using a 10-bp sliding window when the average quality score within the window was below 20, and reads shorter than 50 bp after quality filtering were discarded. Paired-end reads were merged with a minimum overlap of 10 bp, and merged sequences with a mismatch ratio greater than 0.2 in the overlap region were removed. The barcode mismatch was set to 0, and the maximum primer mismatch was set to 2. Chimeric sequences were removed using Usearch against the Gold database with a combined de novo and reference-based strategy. R scripts were run in R v4.0.2. Alpha diversity was calculated using vegan, Tukey's HSD test was performed using multcomp, Bray-Curtis distances and adonis were implemented in vegan, PCoA was performed using ape, and heatmaps were generated using pheatmap. Cohesion, vulnerability, and robustness were calculated from the retained OTU-level co-occurrence networks. Cohesion was used to summarize positive and negative community connectedness, whereas vulnerability and robustness were used to evaluate exploratory changes in network stability after node removal. The numbers of retained nodes and edges for each treatment network are provided in Supplementary Table S1.

Table S1A. Statistical details for the main treatment comparisons shown in Figures 3-5.

Figure panel	Variable	Test	Test statistic	df	p value	Effect size	Letter grouping	Tukey-adjusted pairwise p values
Figure 3a	Chao1 index	One-way ANOVA followed by Tukey's HSD test	F=2.482	2, 6	0.164	η^2=0.453	CK=a; IP=a; AP=a	CK vs IP=0.520; CK vs AP=0.145; IP vs AP=0.562
Figure 3b	ACE index	One-way ANOVA followed by Tukey's HSD test	F=2.103	2, 6	0.203	η^2=0.412	CK=a; IP=a; AP=a	CK vs IP=0.456; CK vs AP=0.186; IP vs AP=0.746
Figure 3c	Shannon index	One-way ANOVA followed by Tukey's HSD test	F=11.905	2, 6	0.008	η^2=0.799	CK=b; IP=b; AP=a	CK vs IP=0.389; CK vs AP=0.036; IP vs AP=0.008
Figure 3d	Observed species	One-way ANOVA followed by Tukey's HSD test	F=2.446	2, 6	0.167	η^2=0.449	CK=a; IP=a; AP=a	CK vs IP=0.705; CK vs AP=0.152; IP vs AP=0.413
Figure 4a	Group distance	One-way ANOVA followed by Tukey's HSD test	F=8.079	2, 15	0.004	η^2=0.519	CK=b; IP=a; AP=a	CK vs IP=0.011; CK vs AP=0.007; IP vs AP=0.969
Figure 4b central PCoA	Bray-Curtis community composition	PERMANOVA using adonis with 999 permutations	pseudo-F=1.716	2, 6	0.003	R^2=0.364	Not applicable	Not applicable

Figure panel	Variable	Test	Test statistic	df	p value	Effect size	Letter grouping	Tukey-adjusted pairwise p values
Figure 4b upper marginal boxplot	PC1 score	One-way ANOVA followed by Tukey's HSD test	F=62.653	2, 6	<0.001	η^2=0.954	CK=a; IP=b; AP=b	CK vs IP<0.001; CK vs AP<0.001; IP vs AP=0.666
Figure 4b right marginal boxplot	PC2 score	One-way ANOVA followed by Tukey's HSD test	F=4.862	2, 6	0.056	η^2=0.618	CK=ab; IP=b; AP=a	CK vs IP=0.340; CK vs AP=0.324; IP vs AP=0.047
Figure 5b	*Methylobacillus* relative abundance	One-way ANOVA followed by Tukey's HSD test	F=12.809	2, 6	0.007	η^2=0.810	CK=a; IP=a; AP=b	CK vs IP=0.461; CK vs AP=0.007; IP vs AP=0.026
Figure 5c	Negative-positive cohesion	One-way ANOVA followed by Tukey's HSD test	F=3.136	2, 6	0.117	η^2=0.511	CK=a; IP=a; AP=a	CK vs IP=0.325; CK vs AP=0.661; IP vs AP=0.106
Figure 5e	Robustness	One-way ANOVA followed by Tukey's HSD test	F=15.364	2, 25	<0.001	η^2=0.551	CK=b; IP=b; AP=a	CK vs IP=0.255; CK vs AP=0.002; IP vs AP<0.001

Table S1B. Co-occurrence network construction parameters.

Treatment	Network level	Correlation method	Correlation cutoff	p-value criterion	Number of nodes (OTU)	Number of edges	Network indices
CK	OTU	Spearman correlation	$\|\rho\|>0.6$	$p<0.05$	184	2609	cohesion, vulnerability, robustness
IP	OTU	Spearman correlation	$\|\rho\|>0.6$	$p<0.05$	192	3358	cohesion, vulnerability, robustness
AP	OTU	Spearman correlation	$\|\rho\|>0.6$	$p<0.05$	176	2589	cohesion, vulnerability, robustness
